# Relative efficiencies of peptidylarginine deiminase 2 and 4 in generating target sites for anti-citrullinated protein antibodies in fibrinogen, alpha-enolase and histone H3

**DOI:** 10.1371/journal.pone.0203214

**Published:** 2018-08-30

**Authors:** Dres Damgaard, Mandar Bawadekar, Ladislav Senolt, Allan Stensballe, Miriam A. Shelef, Claus H. Nielsen

**Affiliations:** 1 Institute for Inflammation Research, Center for Rheumatology and Spine Diseases, Copenhagen University Hospital, Rigshospitalet, Copenhagen, Denmark; 2 Section for Periodontology, Microbiology and Community Dentistry, Department of Odontology, Faculty of Health and Medical Sciences, University of Copenhagen, Copenhagen, Denmark; 3 Department of Medicine, University of Wisconsin, Madison, Wisconsin, United States of America; 4 Institute of Rheumatology and Department of Rheumatology, 1st Faculty of Medicine, Charles University, Prague, Czech Republic; 5 Department of Health Science and Technology, Aalborg University, Aalborg, Denmark; 6 William S. Middleton Memorial Veterans Hospital, Madison, Wisconsin, United States of America; Duke University School of Medicine, UNITED STATES

## Abstract

**Objective:**

Peptidylarginine deiminase 2 (PAD2) and PAD4 are expressed in the synovium of rheumatoid arthritis (RA) patients and catalyze citrullination of arginine residues in proteins targeted by anti-citrullinated protein antibodies (ACPAs). Little is known about the relative importance of PAD2 and PAD4 in generating citrullinated self-antigens. Here we investigate the ability of PAD2 and PAD4 to generate citrullinated targets for ACPAs in four human proteins.

**Methods:**

Synovial fluid (SF) and plasma were collected from 42 RA patients. Human fibrinogen, human alpha-enolase (ENO1), human histone H3, and human serum albumin (HSA) were citrullinated *in vitro* by PAD2 or PAD4. The total degree of citrullination was determined using the anti-modified citrulline approach. Antibody binding to native and citrullinated proteins was measured by ELISA.

**Results:**

ACPAs within pooled SF from multiple RA patients reacted equally well with, and cross-reacted with, PAD2- and PAD4-citrullinated fibrinogen. ACPAs from most individual patient SF and plasma samples bound equally well to PAD2- and PAD4-citrullinated fibrinogen or ENO1. When histone H3 was used as target, PAD4 was generally superior in generating epitopes recognized by ACPAs. No binding to citrullinated HSA was observed.

**Conclusion:**

In most patients, PAD2 and PAD4 are equally efficient in generating citrullinated target sites for ACPAs in fibrinogen and ENO1. The binding of autoantibodies to histone H3 was generally higher after citrullination with PAD4 than with PAD2. Citrullinated HSA is not a target for ACPAs.

## Background

Production of anti-citrullinated protein antibodies (ACPAs) characterizes a subgroup comprising around 70% of patients with rheumatoid arthritis (RA) [1–3]. In this subset, disease is strongly associated with HLA-DRB1 molecules containing a shared amino acid composition (shared epitope) in the peptide binding groove [3,4]. With a relatively poor prognosis, more erosive disease, and association with environmental factors such as smoking, ACPA-positive RA differs from ACPA-negative RA [4–8]. ACPA-positive RA is usually diagnosed as a positive antibody reaction with a synthetic cyclic citrullinated peptide (CCP), the anti-CCP test. ACPAs are highly specific for RA, even though the presence of citrullinated proteins in the synovium also occurs in other inflammatory diseases [9].

Citrullination (deimination) refers to the posttranslational conversion of peptidylarginine into peptidylcitrulline as catalyzed by peptidylarginine deiminases (PADs), of which five isoforms exist [10]. PAD2 and PAD4 are expressed in RA synovium and their presence correlates with inflammatory markers [11–13]. Among the best described PAD substrates is fibrinogen, which is present in the synovium of RA patients [14–18] together with numerous other citrullinated self-antigens including vimentin, alpha-enolase (ENO1), collagen type II and histones, all of which have proposed pathogenic relevance [19–24]. PAD2 seems less restricted by the amino acid composition surrounding the acceptor arginine residue than PAD4 [25]. According to some investigators, PAD2 citrullinates more arginine residues in fibrinogen than PAD4 [26], while others have reported that the two PAD isoforms citrullinate a similar number of sites [27]. The two isoforms show similar dependency on calcium [27,28], reducing conditions [29,30] and pH [27], and their cellular expression and function varies between different leukocyte populations [31–33]. It is unclear if either isoform dominates in the generation of citrullinated self-protein sites targeted by ACPAs. A recent study of 12 ACPA-positive RA patients showed that in highly diluted serum, higher ACPA reactivity was found against fibrinogen citrullinated by PAD4 than against fibrinogen citrullinated by PAD2 [34].

Isolated ACPAs react with citrullinated sites on multiple proteins[35,36], but non-overlapping reactivities have also been demonstrated [35]. Several studies have examined the fine specificity of ACPAs using citrullinated peptides, including some derived from fibrinogen, ENO1, collagen type II and pro-filaggrin, and ACPAs from some individuals bind to several sites whereas others react with only a single site [21,36,37]. PAD2-specific and PAD4-specific inhibitors are currently under development for therapeutic use, and studies providing a better understanding of their relative efficiency in generating citrullinated autoantigens are warranted.

In this study, we aimed to determine the relative contributions by PAD2 and PAD4 to the generation of antigenic determinants in full-length proteins targeted by ACPAs. To this end, we examined the binding of autoantibodies from RA synovial fluid (SF) and plasma from 42 patients to *in vitro* citrullinated fibrinogen, ENO1, histone H3 and human serum albumin (HSA), as well as to the native proteins.

## Methods

### Plasma and synovial fluid from RA patients

SF samples were obtained during knee joint aspiration from 10 anti-CCP-negative (median anti-CCP: 3.6 U/ml [interquartile range: 2–11.4 U/ml]) and 32 anti-CCP-positive (878 U/ml; [136–1353] U/ml) RA patients fulfilling the American College of Rheumatology criteria [38] and characterised with respect to disease-activity, as assessed by DAS28. The majority of the SF samples were also used in a recent study [12]. All SF samples were centrifuged at 1900 *g* for 10 min to remove cells and debris and stored at -80°C until use. Plasma from all patients but four was isolated from peripheral venous blood from the same patients at the time of arthrocentesis. The study was approved by the local ethics committee of the Institute of Rheumatology in Prague (No. 3294/2012), Czech Republic, and written informed consent was obtained from all patients.

Serum anti-CCP antibodies and IgM-rheumatoid factor were determined by standard ELISA kits (TestLine Clinical Diagnostics, Brunn, Czech Republic). Serum CRP was measured using an immuno-turbidimetric technique with an Olympus biochemical analyser, model AU 400 (Olympus, Tokyo, Japan) and SF leukocytes were counted using the Iris IQ200 (Beckman Coulter, CA, USA) analyzer.

### In vitro citrullination

Recombinant human PAD2 and PAD4 were obtained from Cayman Chemicals (Michigan, USA). Plasminogen-depleted fibrinogen (Merck Life Science, Hellerup, Denmark) was diluted to 0.5 mg/mL in citrullination buffer (100 mM Tris-HCl, 1 mM DTT, 5 mM CaCl_2_, pH 7.5), and incubated for 3 hours at room temperature (RT) with or without various concentrations of PAD2 or PAD4. ENO1 (Antibodies-online, Aachen, Germany) and histone H3 (Sigma-Aldrich, Copenhagen, Denmark) was diluted to 0.15 mg/mL in citrullination buffer, and HSA (Statens Serum Institute, Copenhagen, Denmark) at 0.5 mg/mL, with or without 2 μg PAD per mg protein.

### ELISA for anti-citrullinated protein antibodies

Citrullinated or non-citrullinated proteins were diluted in coating buffer (30 mM Na_2_CO_3_, 70 mM NaHCO_3_, pH 9.6) and used for coating microtiter plates (Nunc, Roskilde, Denmark) (100 μL/well) overnight at 4°C. Plates were coated with fibrinogen at 5 μg/mL, whereas the other proteins were used at a concentration of 10 μg/mL. To compensate for content of PAD2 or PAD4 in citrullinated samples, the enzymes were added to negative control wells devoid of other proteins. Wells were washed thrice in PBS, 0.05% Tween-20, pH 7.4, and blocked in dilution buffer (PBS, 0.05% Tween-20, 2% bovine serum albumin). Plasma or SF was diluted in dilution buffer, added to the wells, and incubated for 2 hours at room temperature (RT), including a positive control, consisting of a SF pool, used for normalization. For determination of ACPA cross-reactivity, SF was pre-incubated with the citrullinated proteins at 20, 100 and 200 μg/mL for 30 min before addition to the plates. After three washes, wells were incubated with 100 μL horseradish peroxidase (HRP)-conjugated polyclonal rabbit anti-human immunoglobulin G (IgG) antibodies (P0214, Dako, Glostrup, Denmark) diluted 1:1000 in washing buffer for 1 hour. Finally, the plates were washed three times in washing buffer and incubated with 0.4 mg/mL o-phenylene-diamine (OPD; Kem-En-Tec; Taastrup, Denmark) in developing buffer (35 mM citric acid, 65 mM Na_2_PO_4_, pH 5.0). After 10 min, the colour reaction was stopped with 1.0 M H_2_SO_4_, and optical density (OD) was measured at 490–650 nm using the SPECTROstar nano Microplate Reader (BMG Labtech). Data were processed using the MARS software (BMG Labtech). To determine the specific binding of ACPAs, OD values obtained from coating with native proteins were subtracted from the corresponding values obtained with the citrullinated protein.

### Western blotting

Gel-electrophoresis was performed with NuPAGE 10% Bis-Tris gels (Invitrogen, CA, USA) using the NuPAGE® system (Invitrogen) according to the manufacturer's recommendations. A second gel was stained in parallel with Coomassie Blue. Fibrinogen was loaded at 80 μg/mL in reducing SDS buffer. Proteins were electro-blotted onto pre-activated Immobilon®-FL polyvinylidene difluoride membranes (PVDF; Millipore) by tank-blotting. The membrane was blocked in Odyssey Blocking buffer (LI-COR, Cambrigde, UK) for 1 hour under gentle shaking at RT. An SF pool was diluted 1:100 in blocking buffer including 0.2% Tween-20 for 2 hours under gentle shaking at RT. The membrane was washed three times in TBS 0.2% Tween-20 and incubated with rabbit anti-human IgG antibodies (A0424, Dako) for 1 hour gently shaking at RT before another wash cycle. Finally, the membrane was incubated 1:15,000 with IRDye 800CW-conjugated goat anti-rabbit IgG antibody (LI-COR) in blocking buffer including 0.2% Tween-20 for 1 hour under gentle shaking at RT. After a final wash cycle, the membrane was washed in TBS and left for 1 hour in dark to dry. Membrane was imaged using the Odyssey Fc infrared scanner (LI-COR).

Gel electrophoresis, western blotting, and densitometry to detect and quantify total protein using brilliant blue G colloidal solution (Millipore-Sigma, St. Louis, USA) and protein citrullination using the anti-citrulline (modified) detection kit (Millipore-Sigma) were performed as previously described [39].

### Statistics

Wilcoxon matched-pairs signed rank test and Spearman’s correlation test were determined using GraphPad Prism 7.02 (GraphPad Software, La Jolla, CA, USA). Statistical comparison between PAD2- and PAD4- citrullinated proteins in AMC-blots were made using a paired t-test. P-values < 0.05 were considered statistically significant.

## Results

**Fig 1:**
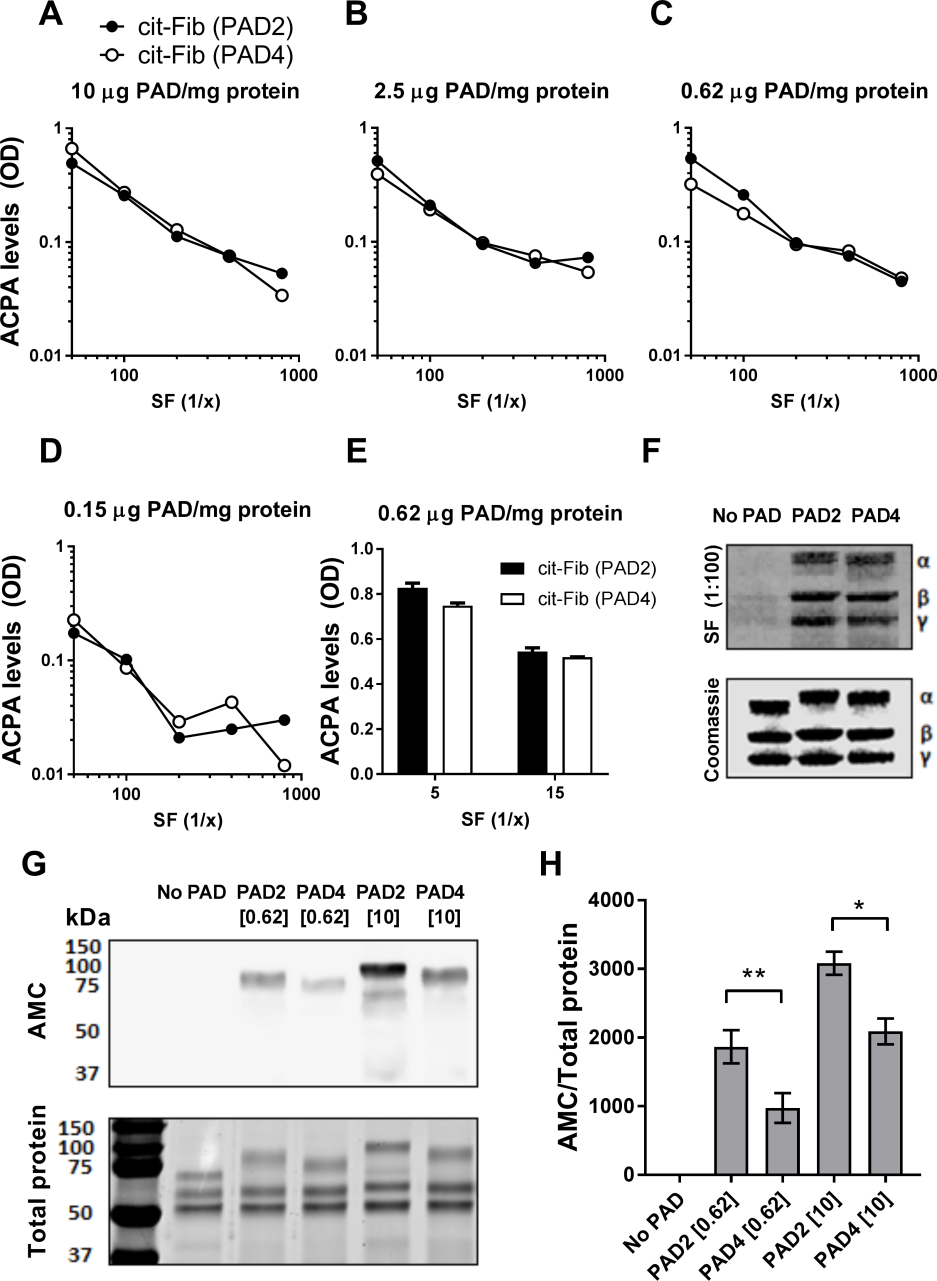
ACPA reactivity with PAD2- and PAD4-citrullinated fibrinogen. **(A-D)** Microtiter plates were coated with native fibrinogen or fibrinogen citrullinated by either PAD2 or PAD4 (cit-Fib) at various concentrations. Pooled synovial fluid (SF) from 6 anti-CCP-positive RA patients was applied at different dilutions ranging from 1:50 to 1:800, and the binding of ACPAs was detected using HRP-anti-human IgG and OPD substrate. Shown are optical density (OD) values from citrullinated minus native fibrinogen. **(E)** The pooled SF was also tested in dilutions of 1:5 and 1:15 using fibrinogen citrullinated by 0.62 μg PAD per mg protein. Average and range of duplicate measurements are shown. **(F)** Western blotting showing the binding of ACPAs from pooled SF (diluted 1:100) to native fibrinogen and fibrinogen citrullinated by PAD2 or PAD4 (0.62 μg PAD per mg protein). Coomassie staining is shown as a loading control. The three bands show α-chain, β-chain and γ-chain of fibrinogen. **(G)** Anti-modified citrulline (AMC) western blot to detect citrullinated fibrinogen, citrullinated by 0.62 or 10 μg PAD per mg protein, respectively, (top) and brilliant blue stained gel to detect total protein (bottom). **(H)** The density of the visible citrullinated protein bands in the AMC blot was normalized to corresponding protein signal. Mean and SEM are shown for triplicate blots with *p<0.05, **p<0.01.

A direct ELISA was used to examine the binding of ACPAs contained in pooled SF from 6 RA patients to native fibrinogen and fibrinogen citrullinated *in vitro* by PAD2 or PAD4. ACPA binding was expressed as net binding, i.e. subtraction of signals obtained with native fibrinogen from signals obtained with citrullinated fibrinogen (Fig 1). ACPAs bound equally well to PAD2-citrullinated and PAD4-citrullinated fibrinogen, irrespective of the PAD concentration and SF dilution used (Fig 1A–1E). This observation was confirmed by Western blotting, which showed that ACPAs bound to all three chains of fibrinogen (Fig 1F).

The preparations of PAD used in these experiments citrullinated fibrinogen efficiently over a wide range of enzyme:substrate ratios (Fig 1G). When the amount of citrullinated fibrinogen was normalized to total protein (Fig 1H), PAD2 appeared to citrullinate fibrinogen more efficiently than PAD4 (Fig 1H).

**Fig 2:**
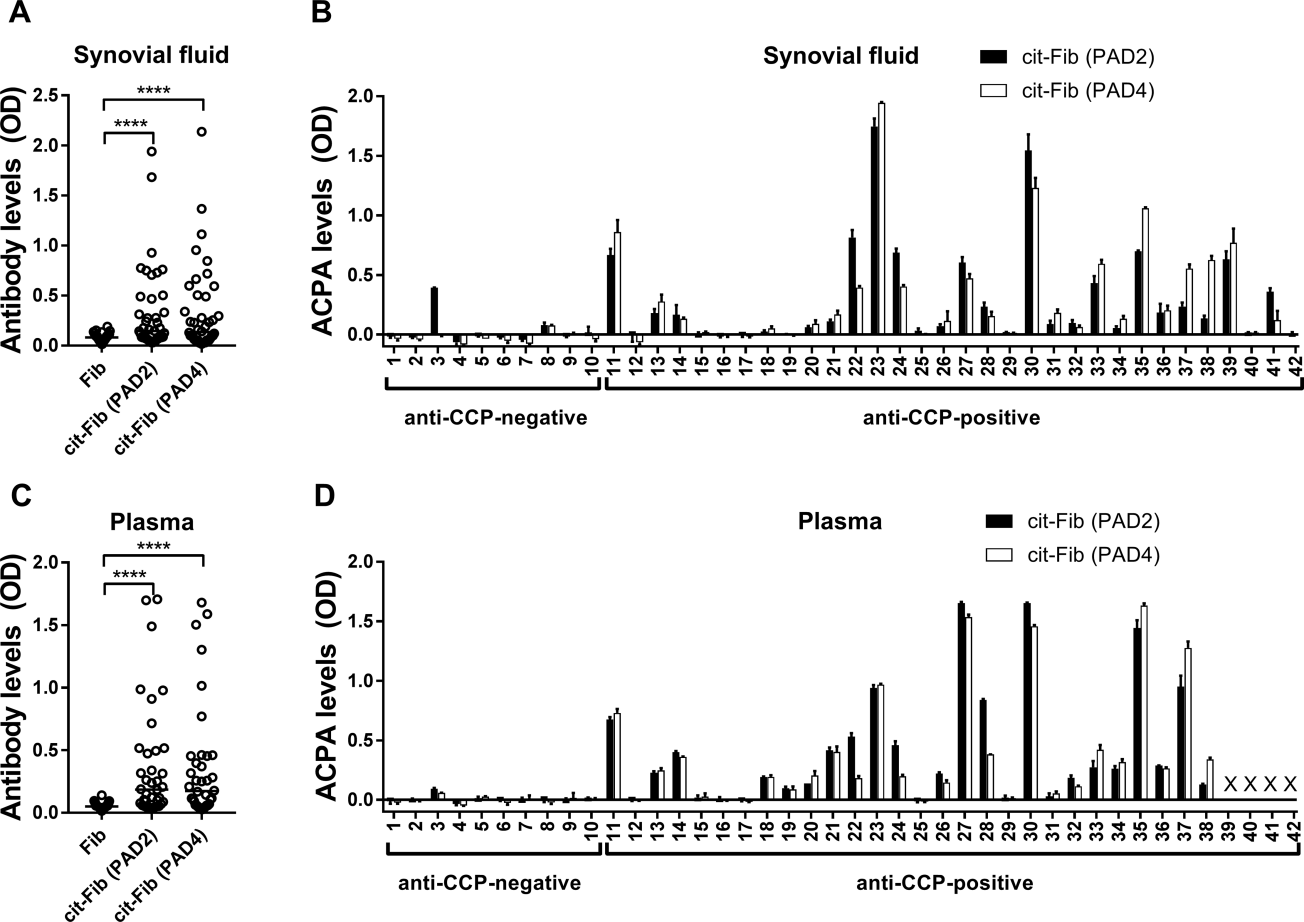
Anti-citrullinated fibrinogen autoantibodies in SF and plasma from individual RA patients. IgG autoantibodies against native and citrullinated fibrinogen were measured in **(A, B)** SF (diluted 1:50) and **(C, D)** plasma (diluted 1:50) from 32 anti-CCP-positive and 10 anti-CCP-negative RA patients using a direct ELISA based on microtiter plates coated with native fibrinogen or fibrinogen citrullinated by either PAD2 or PAD4 (cit-Fib) at 0.62 μg PAD per mg protein. Shown are **(A, C)** raw optical density (OD) values for all patients, and **(B, D)** net OD values all patients, adjusted for binding to native fibrinogen. Each data point represents duplicate measurements. ****p<0.0001.

Forty-two SF samples from RA patients were examined individually for autoantibody reactivity against native, PAD2-citrullinated and PAD4-citrullinated fibrinogen (Fig 2A). All samples contained very little reactivity against native fibrinogen and negligible reactivity with PAD2 or PAD4 (S1 Fig). ACPAs from the majority of anti-CCP-positive samples reacted with similar efficiency against PAD2-citrullinated and PAD4-citrullinated fibrinogen (Fig 2B). However, two patients showed more than two-fold higher ACPA-binding to PAD2-citrullinated than to PAD4-citrullinated fibrinogen (SF# 22 and 41), while two patients showed the opposite pattern (SF# 37 and 38). Notably, 10 of the 32 SF samples from anti-CCP positive patients contained very little or no ACPA-reactivity against fibrinogen citrullinated by either PAD isoform. One SF sample from an anti-CCP-negative patient (#3), on the other hand, contained as much reactivity against PAD2-citrullinated fibrinogen as SF from most anti-CCP-positive patients.

We also tested plasma samples from 38 of the patients, with similar results (Fig 2C and 2D).

### Association between ACPAs and disease activity

The reactivity of ACPAs against fibrinogen citrullinated by PAD2 correlated with patient disease activity, as measured by DAS28-ESR, and with inflammatory burden, measured as serum CRP levels (Table 1). The correlation with DAS28-CRP was not significant, however (p = 0.15 for SF and p = 0.39 for plasma; data not shown). The binding of ACPAs to fibrinogen citrullinated by PAD4 correlated with serum CRP levels and there was a trend towards correlation with DAS28-ESR. As expected, the levels of antibodies against citrullinated fibrinogen correlated strongly with serum anti-CCP levels, while no correlation was found with IgM rheumatoid factor levels or leukocyte concentration in SF.

**Table 1:** Correlation between ACPA levels and disease parameters in 32 anti-CCP-positive RA patients.

	DAS28-ESR*	CRP (mg/l)	Anti-CCP (U/ml)	RF	Leukocyte/μl
**Anti-cit-Fib (PAD2) SF**	**r**_**s**_ **= 0.37 p = 0.042**	**r**_**s**_ **= 0.36 p = 0.041**	**r**_**s**_ **= 0.58 p = 0.002**	r_s_ = 0.27 p = 0.13	r_s_ = 0.02 p = 0.90
**Anti-cit-Fib (PAD4) SF**	r_s_ = 0.34 p = 0.06	**r**_**s**_ **= 0.41 p = 0.019**	**r**_**s**_ **= 0.56 p = 0.001**	r_s_ = -0.18 p = 0.31	r_s_ = 0.11 p = 0.55
**Anti-cit-Fib (PAD2) Plasma**	r_s_ = 0.33 p = 0.10	r_s_ = 0.33 p = 0.083	**r**_**s**_ **= 0.58 p = 0.001**	r_s_ = 0.31 p = 0.11	r_s_ = -0.17 p = 0.39
**Anti-cit-Fib (PAD4) Plasma**	r_s_ = 0.34 p = 0.085	**r**_**s**_ **= 0.39 p = 0.041**	**r**_**s**_ **= 0.66 p = 0.0001**	r_s_ = 0.27 p = 0.16	r_s_ = -0.10 p = 0.61

Cit-Fib (PAD2): Fibrinogen citrullinated by PAD2, cit-Fib (PAD4): Fibrinogen citrullinated by PAD4, SF: synovial fluid, CRP: C-reactive protein, ESR: Erythrocyte sedimentation rate, RF: Rheumatoid factor. Spearman’s correlation coefficients (r_s_) and levels of significance are shown.

*DAS28-ESR data was missing for four patients.

### Autoantibody binding to native and citrullinated ENO1, histone H3 and HSA

**Fig 3:**
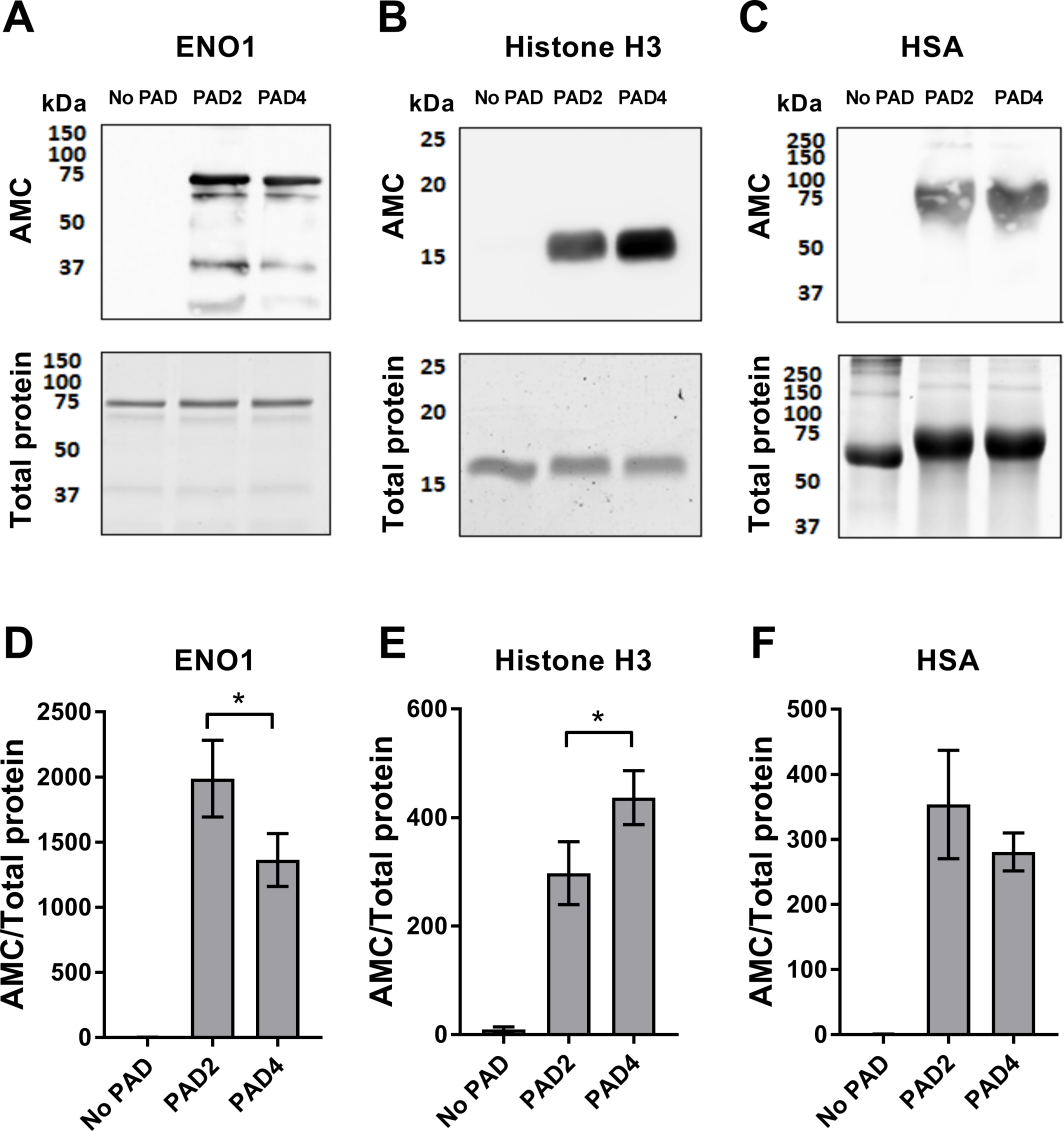
*In vitro* citrullination of ENO1, histone H3 and HSA by PAD2 and PAD4. **(A)** ENO1, **(B)** histone H3 and **(C)** HSA were citrullinated with PAD2 and PAD4 (2 μg PAD per mg protein). Western blots (upper panels) show citrullination, as detected by the AMC kit, and gel electrophoresis followed by brilliant blue staining (lower panels) shows total protein. **(D-F)** The density of the visible bands corresponding to citrullinated protein in the AMC blot was normalized to the total protein signal. Mean and SEM are shown for triplicate blots, *p<0.05.

To examine the binding of ACPAs to proteins other than fibrinogen, ENO1, histone H3 and HSA were citrullinated by PAD2 and PAD4 (Fig 3A–3C). PAD2 citrullinated ENO1 more efficiently than PAD4, while histone H3 was most efficiently citrullinated by PAD4.

**Fig 4:**
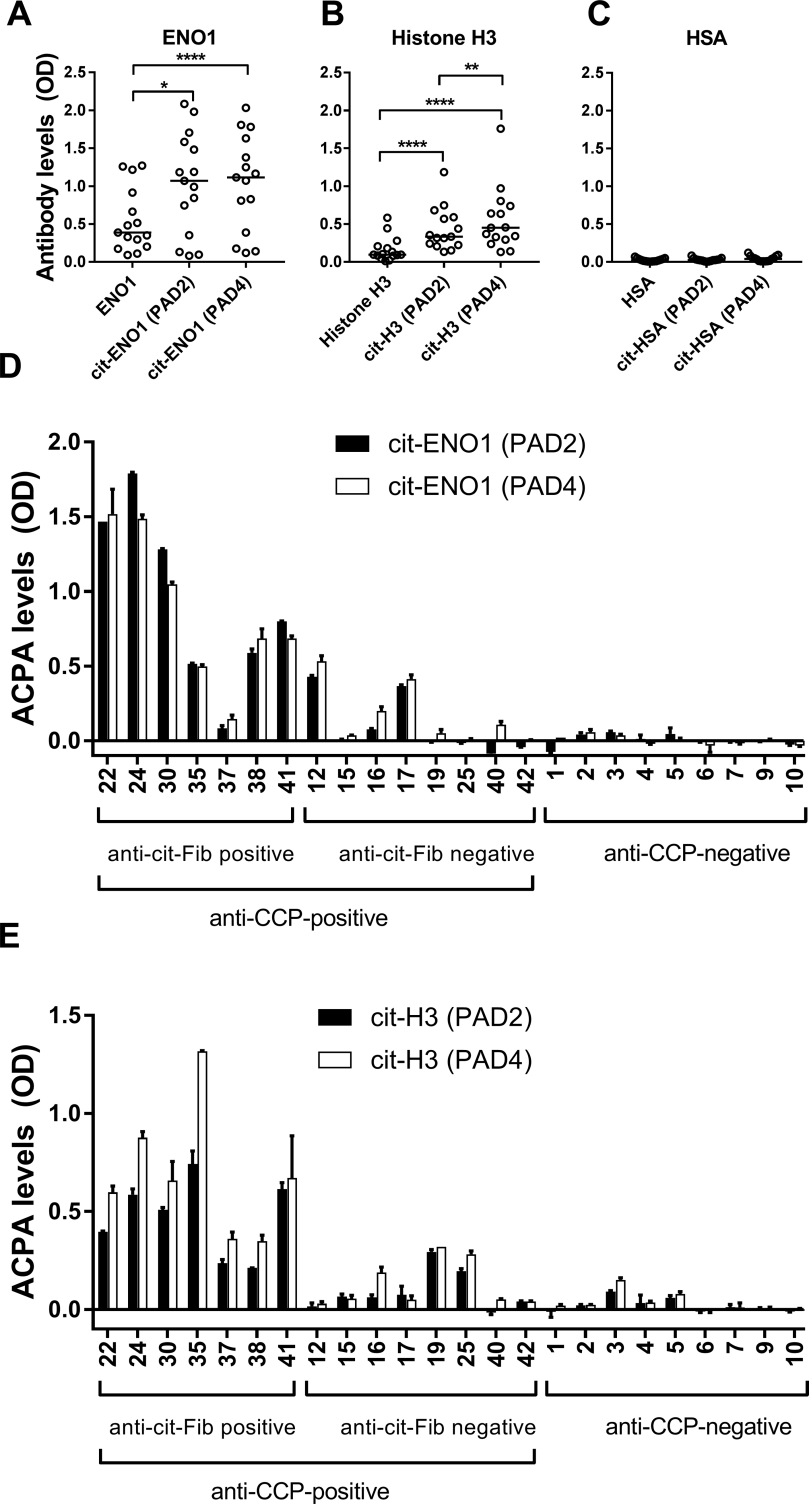
Binding of antibodies from RA patients to citrullinated and native proteins. Microtiter plates were coated with native protein or protein citrullinated by either PAD2 or PAD4 (2 μg PAD per mg protein), and SF samples from 15 anti-CCP-positive RA patients were applied at a 1:50 dilution. Binding of IgG antibodies to native or citrullinated **(A)** ENO1, **(B)** histone H3 and **(C)** HSA are shown as an average of duplicate optical density (OD) measurements, and horizontal bars represent median values. **(D)** ACPA binding to citrullinated ENO1 and **(E)** histone H3 was calculated for the 15 anti-CCP-positive RA patients and additional 9 anti-CCP-negative RA patients. Shown are averages and range of duplicate OD measurements, after subtraction of signals obtained for native protein.

SF samples from 15 anti-CCP-positive RA patients were examined for antibody reactivity against ENO1, histone H3 and HSA (Fig 4A–4C). A remarkably high antibody-reactivity was observed against native ENO1 (Fig 4A) and, in some cases, against native histone H3 (Fig 4B). No detectable binding to native HSA was observed (Fig 4C).

The binding of SF autoantibodies was increased for citrullinated ENO1 and histone H3, compared to the native proteins (Fig 4A and 4B), while citrullination of HSA did not cause an increase in antibody binding (Fig 4C). Binding to ENO1 citrullinated by PAD2 or PAD4 was comparable, but in contrast, ACPAs showed preferential binding to histone H3 citrullinated by PAD4 (Fig 4B).

ACPA binding to the citrullinated proteins was calculated for the 15 anti-CCP-positive and 9 anti-CCP negative RA patients. Notably, some patients who had no antibody reactivity against citrullinated fibrinogen showed reactivity against citrullinated ENO1 or histone H3 (Fig 4D and 4E).

More binding to histone H3 was generally observed after citrullination with PAD4 than with PAD2 (Fig 4E). In general, patients with high reactivity against one protein also showed high reactivity against the other proteins, but exceptions were observed, e.g. #19 and #25 who were only positive for citrullinated histone H3.

### Determination of ACPA cross-reactivity

**Fig 5:**
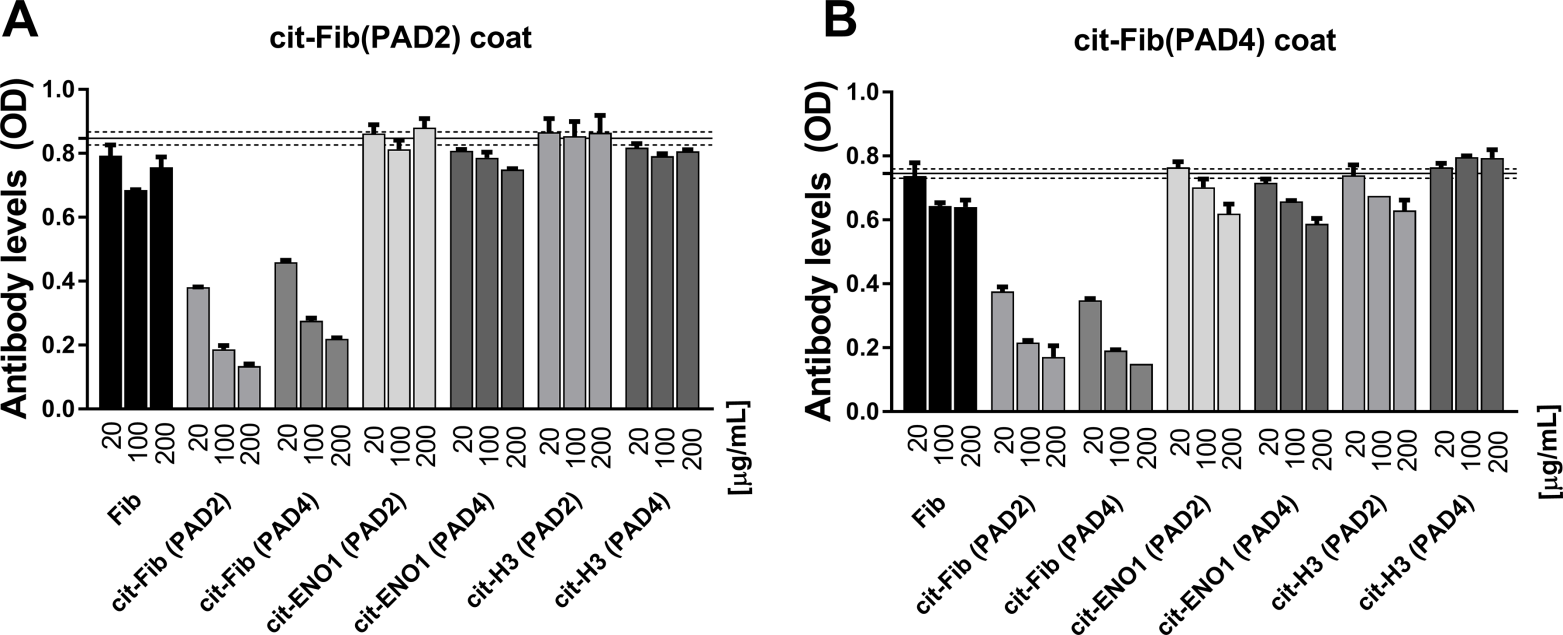
ACPA cross-reactivity assessed by competitive ELISA. Microtiter plates were coated with fibrinogen citrullinated by either **(A)** PAD2 or **(B)** PAD4 (2 μg PAD per mg protein). A pool of SF from three RA patients, diluted 1:50, was pre-incubated with increasing concentrations of native fibrinogen, fibrinogen citrullinated by PAD2 (cit-Fib(PAD2)) or by PAD4 (cit-Fib(PAD4)), ENO1 citrullinated by PAD2 (cit-ENO1(PAD2)) or by PAD4 (cit-ENO1 (PAD4)), or histone H3 citrullinated with PAD2 (cit-H3(PAD2)) or PAD4 (cit-H3(PAD4)) for 30 minutes before addition to the plates. Binding signals obtained in the absence of added protein (n = 7) are shown as mean (solid line) and 95% CI (dotted line). The subsequent binding of IgG antibodies within the SF pool to **(A)** PAD2-citrullinated fibrinogen or **(B)** PAD4-citrullinated fibrinogen, as determined by ELISA, are shown as mean ± SEM of duplicate measurements.

To assess cross-reactivity between ACPAs directed against PAD2- and PAD4-citrullinated fibrinogen, we examined the ability of PAD2-citrullinated fibrinogen to compete for ACPA-binding against PAD4-citrullinated fibrinogen and vice versa. As shown in Fig 5A and 5B, incubation of SF with fibrinogen citrullinated by one PAD isoform strongly inhibited the binding of IgG antibodies to the other isoform, indicating that ACPAs generally do not discriminate between fibrinogen citrullinated by PAD2 and PAD4.

In contrast, citrullinated ENO1 and citrullinated histone H3 showed little or no ability to compete with citrullinated fibrinogen for ACPAs, irrespective of whether PAD2 or PAD4 was used for citrullination (5A and 5B), suggesting a high degree of antigen-specificity. In agreement, fibrinogen citrullinated by PAD2 or PAD4 showed modest or no ability to compete with citrullinated ENO1 or histone H3 for ACPAs (data not shown).

## Discussion

PAD2 and PAD4 are present in the synovium of RA patients [11,40], but their individual roles in generation of citrullinated self-antigens targeted by ACPAs is poorly understood. We have here examined the binding of ACPAs to three proteins (fibrinogen, ENO1 and histone H3) known to be antigens in RA, and to HSA, as a protein not reported to be antigenic, following *in vitro* citrullination with PAD2 or PAD4.

When fibrinogen was used as antigen, autoantibodies from pooled SF from RA patients reacted equally well with PAD2- and PAD4-citrullinated fibrinogen, at any SF dilution employed and at any PAD concentration used for citrullination. These findings are not in complete agreement with the findings of Blachére et al., who found that at high dilutions of serum (1:250 or more), ACPAs bound better to PAD4-citrullinated fibrinogen than to PAD2-citrullinated fibrinogen [34]. We used 1:50 dilutions of SF and plasma as standard, but observed the same pattern at higher dilutions. Differences in the results of Blachére et al. and ours may also have been caused by differences in the citrullination procedure [34], but both studies showed a higher degree of citrullination of fibrinogen by PAD2 than by PAD4. Great degree of overlap between fibrinogen sites citrullinated by PAD2 and PAD4 have been demonstrated, although sites targeted by one PAD isoform, specifically, have also been described [25,27]. Since PAD2-citrullinated fibrinogen competitively inhibited ACPA-binding to PAD4-citrullinated fibrinogen and vice versa in this study, ACPAs seemingly do not show a generalized preferential binding to fibrinogen citrullinated by either isoform. Moreover, citrullinated histone H3 and citrullinated ENO1 competed poorly with citrullinated fibrinogen for ACPA binding and vice versa, supporting the notion that ACPAs are predominantly antigen-specific. Using citrullinated fibrinogen and peptides derived from ENO1 and collagen type II, Snir et al. also found no or modest ACPAs cross-reactivity [36]. However, another study found that citrullinated peptides from filaggrin inhibited binding to intact citrullinated fibrinogen, indicating that filaggrin and fibrinogen contain common epitopes [41]. Of note, we did not observe ACPA binding to citrullinated HSA, indicating that not all citrullinated proteins are targeted by ACPAs.

When SF samples from individual patients were examined, ACPAs from most patients reacted equally well with fibrinogen citrullinated by PAD2 and PAD4, and the same was observed with citrullinated ENO1. Notably, ACPAs from a few patients bound preferentially to fibrinogen citrullinated by one isoform or the other. There was increased ACPA binding to histone H3 citrullinated by PAD4. This may be explained by higher degree of citrullination by PAD4. Moreover, due to its intranuclear location, PAD4 is thought to be the isoform responsible for citrullination of histone H3 in activated neutrophils [32,42,43]. However, it was shown recently that PAD2 also citrullinates histone H3 upon release from stimulated neutrophils [33]. Both isoforms may thus contribute to generation of target sites for ACPAs in histone H3 *in vivo*.

In this study, the binding of autoantibodies to native fibrinogen was negligible, but relatively high autoantibody reactivity against native ENO1 was present. Some patients also had autoantibodies against native histone H3. Other researchers have demonstrated binding of autoantibodies to native proteins in RA, i.e. ENO1 and heterogeneous nuclear ribonucleoproteins A2/B1 [22,44]. Thus, measurement of antibodies to citrullinated proteins may include signals that are caused by antibody-binding to native epitopes, but seemingly this is not the case for fibrinogen. It is conceivable that breakage of tolerance by citrullinated sites in ENO1 and histone H3 leads to formation of autoantibodies targeting native sites via epitope spreading. We have here focused on antibodies reacting with citrullinated epitopes, specifically.

In accordance with a well-established correlation between anti-CCP levels and disease activity [6,45], we observed a link between high reactivity of ACPAs in SF against fibrinogen citrullinated by either PAD isoform and high disease activity, expressed as DAS28-ESR or circulating CRP levels.

Our study indicates that PAD2 and PAD4 are equally efficient at generating target sites for ACPAs, at calcium concentration and pH comparable to those existing in in SF, where citrullination of fibrinogen presumably takes place [14,17,18]. Although the DTT used here as reducing agent may be stronger than physiological reductants, PAD2 and PAD4 have similar activities at various redox conditions [29], so the results presented here are likely to reflect their relative efficiencies in SF where both isoforms are present [32,40]. Due to differences in intracellular and extracellular localization, on the other hand [32,33,42], PAD2 and PAD4 may still play different roles in the pathogenesis of RA and other diseases.

## Conclusions

PAD2 and PAD4 are both capable generating citrullinated epitopes in fibrinogen, ENO1 and histone H3 recognized by ACPAs, while citrullinated HSA is not a target.

## Supporting information

S1 Fig Influence of anti-PAD antibodies in the measurement of ACPAs reacting with citrullinated fibrinogen. IgG autoantibodies against PAD2- or PAD4-coated plates at the corresponding concentration to what is present in the preparations of citrullinated fibrinogen (3 ng/mL). Shown are optical density (OD) values for all patients. Each data point represents duplicate measurements.
(DOCX)

## Abbreviations

PAD: peptidylarginine deiminase
ACPA: anti-citrullinated protein antibody
RA: rheumatoid arthritis
CCP: cyclic citrullinated peptide
ENO1: alpha.enolase
HSA: human serum albumin
