# Supplementary material for: Relative efficiencies of peptidylarginine deiminase 2 and 4 in generating target sites for anti-citrullinated protein antibodies in fibrinogen, alpha-enolase and histone H3
Source: PLoS One. 2018 Aug 30;13(8):e0203214. doi: 10.1371/journal.pone.0203214 (PMC6117052; doi:10.1371/journal.pone.0203214)
Supplement: S1 Fig — IgG autoantibodies against PAD2- or PAD4-coated plates at the corresponding concentration to what is present in the preparations of citrullinated fibrinogen (3 ng/mL). Shown are optical density (OD) values for all patients. Each data point represents duplicate measurements. (DOCX) [file pone.0203214.s001.docx]

**Fig. S1. Influence of anti-PAD antibodies in the measurement of ACPAs reacting with citrullinated fibrinogen.** IgG autoantibodies against PAD2- or PAD4-coated plates at the corresponding concentration to what is present in the preparations of citrullinated fibrinogen (3 ng/mL). Shown are optical density (OD) values for all patients. Each data point represents duplicate measurements.
